# Young ERC Resuscitation Science Masterclass Journal Club article: the ARREST trial

**DOI:** 10.1016/j.resplu.2026.101287

**Published:** 2026-03-13

**Authors:** Florian Gallob, Emma Bürgstein, Cristina Jorge-Soto

**Affiliations:** a Medical University of Graz, Graz, Austria; b KAGes – Styrian Hospital Association, Graz, Austria; c Research Center for Emergency Medicine, Aarhus University, Aarhus, Denmark; d CLINURSID Research Group, Psychiatry, Radiology, Public Health, Nursing and Medicine Department, Universidade de Santiago de Compostela, Santiago de Compostela, Spain; e Simulation and Intensive Care Unit of Santiago (SICRUS) Research Group, Health Research Institute of Santiago, University Hospital of Santiago de Compostela-CHUS, Santiago de Compostela, Spain

As part of the Young European Resuscitation Council Resuscitation Science Masterclass 2023/24, which was depicted previously, journal club articles are published.^1^ This article focusses on the ARREST trial that was published in the Lancet in 2023. ARREST is the only randomized controlled trial (RCT) investigating the effect of direct transportation to cardiac arrest centers (CACs) after an out-of-hospital cardiac arrest (OHCA).^2^

## Which knowledge gap does this study try to fill out?

The idea of CACs is based on the complexity of post-resuscitation care for survivors after OHCA, which might be met with specifically tailored centers of care.^3^ It seems plausible that heightened exposure combined with strict criteria of available interventions and expertise might be beneficial. However, elongated prehospital time may be associated with worse outcomes and an additional burden on prehospital systems.^4^ Additionally, there might be consequences for hospitals that are bypassed in the concept of a CAC directed model of care (i.e. less exposure leading to less expertise).

Observational studies have suggested a benefit of treatment in CACs in patients after OHCA.^5^, ^6^ However, the inherent risk of bias in observational studies must be highlighted, hence a randomized comparison was needed.

## What was the design of the study?

The ARREST trial was an unblinded RCT, that was conducted in the urban area of London in the United Kingdom. 862 patients with return of spontaneous circulation (ROSC) after OHCA were randomized in a 1:1 ratio to either a transfer directly to the cardiac catheter laboratory of a CAC or to the nearest emergency department (ED).

Noteworthy exclusion criteria were a presumed non-cardiac cause of the arrest and the presence of ST-elevations on the 12-lead electrocardiogram.

## What were the key findings of the study?

The primary outcome of 30-day mortality was neutral with an identical number of 258 events per group (RR 1.00, 95% CI 0·90–1·11; *p* = 0.96). Furthermore, no significant differences were observed in the secondary outcomes, consisting of three-month mortality, neurological status at discharge and after three months (measured by the modified Rankin Scale) and quality of life at discharge (measured by EQ-5D-5L).

While patients with a shockable rhythm were a subgroup with markedly improved outcomes in observational studies, no difference was observed in the ARREST trial.^6^, ^7^, ^8^

## Methodological considerations

Even though the consistently observed benefit in prior observational studies might have been an argument for the strength of evidence, the ARREST trial is in contrast to these results.^5^, ^6^ In these observational studies the play of chance might have been decreased by many included events, however such observational studies are prone to bias.

A particularly relevant bias in observational studies on CACs might be confounding by indication. This bias occurs, when the indication for the exposure under investigation is associated with the outcome, which therefore distorts the association between the exposure and outcome in varying magnitude. Hence, patients with a presumed higher chance of a good outcome might have been transported more often to a CAC, whereas presumably futile cases could have been transported to a local hospital. Methods of adjustments will likely be inadequate to capture the multitude of factors relevant to this decision, which was highlighted in prior publications on confounding by indication in other research areas.^9^, ^10^

Another possible example of confounding by indication might be evident in recent RCTs on intravenous vs. intraosseous access in OHCA, that provided opposing results to prior observational studies as well, possibly because the aforementioned bias was removed by randomization.^11^

## What are the most important strengths and limitations of the study?

ARREST was the first RCT evaluating the benefit of CAC.

The most important limitation in terms of generalizability is the purely urban setting. The additional 7 minutes in prehospital time that were observed in the intervention arm (84 min vs. 77 min) will be surpassed tremendously in more rural areas, if prehospital services were to bypass the nearest ED for a CAC. Notably, in the majority of prior observational data the benefit of a transportation to a CAC was not modified by the transportation duration.^12^, ^13^, ^14^ Due to the increased transportation duration, the impact of the quality of prehospital care must be considered when transferring the results of this RCT to other systems of prehospital care.

Likewise, the differences in capabilities between CAC and non-CAC are not uniform and should always be determined at an individual level, especially with an increased usage of extracorporeal devices. However, in the ARREST trial ECMO was only used in 5 patients in total.

Another important limitation of the ARREST trial is the exclusion of presumed non-cardiac causes for arrests, which led to the exclusion of about one third of all 4019 screened patients. Considering how difficult and imprecise this prehospital adjudication is, this might lead to a non-differential error in the selection of patients and a heterogenous study population.^15^

Additionally, patients without prehospital ROSC were excluded. However, this was a subgroup of added benefit in prior observational studies on CACs.^6^

It is important to highlight that the intervention arm was transported to a cardiac catheter laboratory of a CAC and the control arm to the nearest ED. However, the nearest ED in the control arm could also be in a CAC. In a recently published re-analysis of the ARREST trial three groups were compared based on their transportation destination, namely the ED of a non-CAC, the ED of a CAC and the cardiac catheter laboratory at a CAC. In this analysis it was stated that only 233 of the 413 patients (56%) assigned to the control arm were transported to a non-CAC. Interestingly a benefit was observed for patients transported to a CAC in this re- analysis.^16^ Despite the neutral results of the ARREST intention-to-treat analysis, this suggests a possible benefit of a direct transportation to a CAC in a scenario where the nearest non-CAC would have to be bypassed for a CAC further away. The power to detect any benefit from a direct transportation to a CAC in the intention-to-treat analysis might have been diminished by the inclusion of patients, where the CAC was the next hospital anyway.

Since an inpatient transfer occurred in another 70 patients of the control group (possibly facilitated by the urban setting), only 40% of patients in the control group remained in a non-CAC during their post-ROSC treatment.

## How will the results affect your clinical practice?

In spite of the neutral results of the only RCT on the topic, both a systematic review by ILCOR and the recently published ERC guidelines recommend transportation to a CAC.^17^, ^18^, ^19^ This reflects the persisting uncertainty on the topic.

In our opinion the intervention of a direct transportation to a CAC will inherently differ vastly depending on the study site and the local systems of care, therefore local protocols should be used for transportation targets until further evidence is available.

## What do you see as the next steps in research?

It is important to remember that CACs offer intervention bundles after OHCA. For example, immediate angiography after OHCA in the absence of ST elevation was recently shown to offer no benefit, yet in the ARREST trial it was performed more often in the intervention group and is one of the most prominent hallmarks of CACs.^20^ Hence future studies might want to investigate the impact of specific interventions that are linked to CACs.

The validation of the results from the ARREST trial with the inclusion of a rural trial area, done exclusively in a population where the CAC is not the next destination anyway, is needed to reliably evaluate a possible benefit from direct transportation to a CAC.

## CRediT authorship contribution statement

**Florian Gallob:** Writing – review & editing, Writing – original draft, Visualization, Investigation, Data curation, Conceptualization. **Emma Bürgstein:** Writing – review & editing, Writing – original draft, Visualization, Investigation, Data curation, Conceptualization. **Cristina Jorge-Soto:** Writing – review & editing, Supervision, Conceptualization.

## Declaration of competing interest

The authors declare that they have no known competing financial interests or personal relationships that could have appeared to influence the work reported in this paper.

## Glossary

CAC: cardiac arrest center
ECMO: extracorporeal membrane oxygenation
ED: emergency department
ERC: European Resuscitation Council
ILCOR: International Liaison Committee on Resuscitation
OHCA: out-of-hospital cardiac arrest
RCT: randomized controlled trial
ROSC: return of spontaneous circulation
